# A Rare Case of Digital Ulceration and Gangrene as an Initial Presentation of Systemic Lupus Erythematosus in a Child

**DOI:** 10.7759/cureus.48698

**Published:** 2023-11-12

**Authors:** Shyam Vengala, Varnika Gupta, Vaishnavi Kandukuri, Bala Sai Teja Nuthalapati, Navya Pillikunte Doddareddy, Deepanshu Raj, Mihirkumar P Parmar, Vishal Venugopal

**Affiliations:** 1 Internal Medicine, Government Medical College, Srikakulam, Visakhapatnam, IND; 2 Internal Medicine, Lala Lajpat Rai Memorial Medical College, Meerut, IND; 3 Internal Medicine, Gandhi Medical College, Hyderabad, IND; 4 Internal Medicine, Maheshwara Medical College, Patancheru, IND; 5 Internal Medicine, Bangalore Medical College and Research Institute, Bangalore, IND; 6 Internal Medicine, SSIMS (Shymanuru Shivashankarappa Institute of Medical Sciences) and Research Centre, Davanagere, IND; 7 Internal Medicine, Gujarat Medical Education and Research Society, Vadnagar, IND; 8 Internal Medicine, Bhaarath Medical College and Hospital, Chennai, IND

**Keywords:** child, ulceration, gangrene, autoimmune disorder, sle

## Abstract

Systemic lupus erythematosus (SLE) is a complex autoimmune illness with a wide range of symptoms. Tissue-binding autoantibodies and intricate immune complexes are responsible for the initial damage to organs and cellular structures. Dermatological signs, particularly digital gangrene and ulcers, are uncommon in the context of systemic lupus erythematosus and often appear in the advanced stages of the disease. In this discussion, we present an unusual example of early-onset digital gangrene and ulcers in a young kid with systemic lupus erythematosus. It is unusual because SLE is mostly seen in adult patients, but here the patient is a seven-year-old boy who went to the doctor because he had urticarial rashes all over his body and face, skin desquamation, and sporadic fever episodes. The preliminary evaluation had difficulty separating this presentation from acute urticaria. However, further diagnostic testing and serological analysis confirmed the patient's SLE diagnosis. The distal regions of the fingers developed digital gangrene, ulceration, and vasculitis. Clinical and serological tests were used to confirm the diagnosis. Antinuclear antibodies (ANA), anti-ribonuclear protein (Anti-RNP) antibodies, anti-Smith (Anti-Sm) antibodies, and anti-Sjögren's syndrome-related antigen A (Anti-SS-A) antibodies were all positive in the patient. This example emphasizes the critical need to recognize the unusual and severe signs of SLE in medical practice.

## Introduction

This article was previously posted to the Research Square preprint server on September 25, 2023 [1]. Systemic lupus erythematosus (SLE) is an autoimmune disease that affects a variety of physiological systems, including the integumentary, musculoskeletal, renal, cardiovascular, pulmonary, neurological, hematological, and other organ systems [2]. This disorder occurs when the immune system mistakenly assaults healthy somatic tissues, resulting in a chain reaction of inflammatory reactions and a variety of clinical symptoms [3]. SLE is distinguished by alternating episodes of exacerbation and remission, and its influence on a person's constitution is strikingly variable. SLE may occasionally manifest with atypical or less familiar clinical characteristics in addition to traditional signs such as fatigue, cutaneous eruptions, and arthralgia. Notably, digital ulcers, which have long healing times and persistent pain, may occasionally present as the first sign of SLE in approximately 30% of patients each year [4]. Lupus has phenotypic and genotypic variability, making diagnosis and treatment difficult [5]. The pathogenesis of digital ulcers is thought to be impaired blood perfusion to the digits, which can be caused by vascular inflammation, endothelial injury, or simultaneous Raynaud's phenomenon, which has been linked to SLE [6]. In the current case, we are presented with a clinical scenario involving a seven-year-old male youngster who first arrived with digital ulcers. Positive antinuclear antibodies (ANA), anti-ribonuclear protein (anti-RNP), anti-Smith (anti-SM), and anti-Sjögren's syndrome-related antigen A (anti-SS-A) antibody titers were present along with this presentation.

## Case presentation

**Figure 1:**
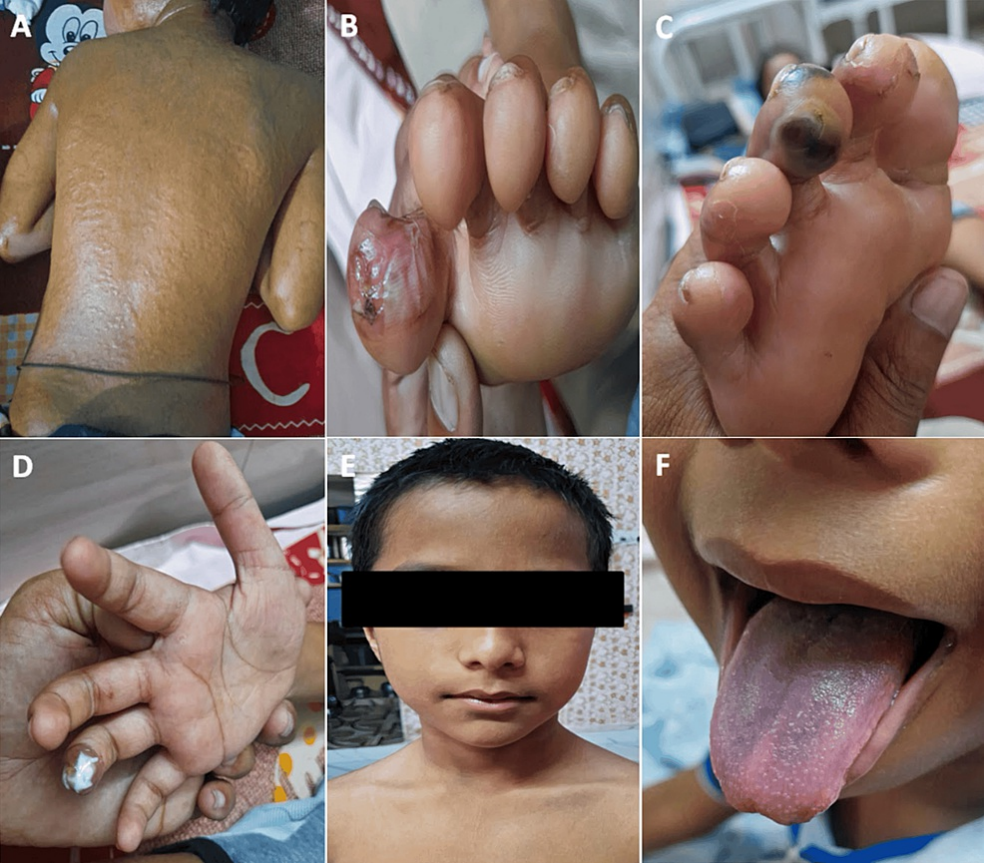
Presented clinical features (A) Patients showing diffuse rash on the dorsal trunk; (B) patient showing ulceration in the left great toe; (C) digital gangrene in the left third toe; (D) digital gangrene in the right fourth finger; (E) frontal baldness in the patient; and (F) cyanosis of the tongue.

The case pertains to a seven-year-old male child of Dravidian descent who presents with a three-week history of diffuse rash spanning his dorsal trunk (Figure 1A) and facial regions, accompanied by skin desquamation on the palms. Additionally, the patient exhibits digital pain, ulceration on the distal extremities (Figure 1B), and blackish discoloration and gangrenous features of the left third toe (Figure 1C) and right fourth finger (Figure 1D). Concurrently, the child experienced recurring fever episodes. Physical examination revealed frontal baldness (Figure 1E) and a bluish discoloration of the tongue (Figure 1F), while all vital signs remained within normal parameters. Notably, there is no reported photosensitivity. Peripheral pulses were palpable, and the Sexual Maturity Rating (SMR) aligns with the patient's age with a normal genital examination. The remaining aspects of the physical evaluation revealed no anomalies.

The results of the laboratory tests show that the patient's hemoglobin level is 13.4 g/dl (normal value (NV): 11.5-15.5), his platelet count is 340,000 cells/mm^3^ (NV: 100,000-400,000), and his total white blood cell count is 15,300 cells/mm^3^ (NV: 5,000-10,000). Anti-nuclear, anti-Sm, anti-SS-A, and anti-RNP antibodies are among the immunological tests that yield positive findings. On the other hand, antibodies against Sjögren's syndrome type B (Anti-SSB), anti-neutrophilic cytoplasmic antibodies (ANCA), and anti-mitochondrial M2 antibody (Anti-M2) show adverse effects. Table 1 displays comprehensive ANA profile data. The results of the upper and lower limb Doppler ultrasonography were normal. Also, the patient’s family has no history of similar illnesses.

**Table 1:** Detailed ANA profile results ANA: antinuclear antibodies, ANCA: anti-neutrophilic cytoplasmic antibodies, anti-SSB: anti-Sjögren's syndrome type B, Anti-M2: anti-mitochondrial M2 antibody, AMA M2: anti-mitochondrial M2 antibody. Antibodies exhibit negative outcomes.

Antigen	Intensity	Class
Ribonucleoprotein (RNP)	34	++
Smith (Sm)	27	++
Sjögren's syndrome-related antigen A (SS-A) (Ro)	12	+
Ro 52 recombinant	4	0
Sjögren's syndrome-related antigen B (SS-B) (La)	1	0
Scleroderma and a 70-kD extractable immunoreactive fragment (70 sci)	3	0
Polymyositis and scleroderma (PM Sci 100)	3	0
Jo-1	0	0
Centromere B	2	0
Double-stranded DNA (dsDNA)	2	0
Proliferating cell nuclear antigen (PCNA)	0	0
Nucleosomes	0	0
Histones	0	0
Ribosomal protein	3	0
AMA M2	3	0

A series of tests were performed to distinguish the vascular etiology underlying the cutaneous symptoms. Prothrombin time (PT), cryoglobulin analysis, anti-cardiolipin antibodies, anti-beta2 glycoprotein antibodies, and lupus anticoagulant assessment were the tests performed; all of the results were negative. The lipid profile of the patient was within normal limits. Additionally, negative findings from serological tests for HIV, hepatitis B, and hepatitis C were obtained.

There was no history of infection, trauma, Raynaud phenomenon, diabetes mellitus, or chemical exposure. There was no history of thromboembolic incidents in the family. Based on the American College of Rheumatology's (ACRC) criteria, the youngster was identified as having SLE, gangrene, and digital ulcers.

Management

**Figure 2:**
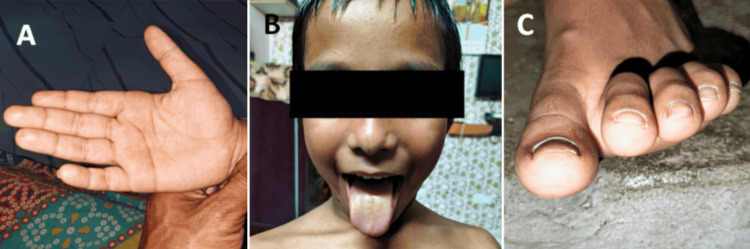
Treatment results (A) Improvement of skin lesions and cyanosis of the tongue after corticosteroid administration and improvement of digital gangrene in the right fourth digit; (B) improved cyanosis of tongue; and (C) improvement of ulceration of left great toe.

Prednisone (2 mg/kg/day) was administered to the patient for a period of three months. Over the following three months, the prednisone dosage was gradually lowered to 5 mg/day. Steroid treatment, silver sulfadiazine, and hydroxyzine were effective in treating the cutaneous symptoms and digital ulcers [7]. After receiving corticosteroids, the skin lesions and tongue cyanosis improved. Additionally, the digital gangrene in the right fourth digit (Figure 2A) and tongue cyanosis (Figure 2B) improved, and the ulcers on the left great toe (Figure 2C) improved.

The child was followed every three months for a year, and gradual remission was observed with no recurrence of any cutaneous or thromboembolic events.

## Discussion

As common as they are in connective tissue disorders, the development of digital ulcers and gangrene in SLE is exceedingly rare, even rarer when they present as a primary manifestation. Dubois and Arteberry [8] and Alarcon-Segovia and Osmundson [9] were the first to report SLE with distal gangrene. In a United Kingdom (UK) study involving patients with SLE, 12% of the 179 patients with skin involvement reported having cutaneous vasculitis [10].

This patient has no history of SLE or any manifestation of connective tissue or an inflammatory disorder. Critical peripheral ischemia (CPI) is defined as pain, pallor, ulceration, and necrosis of the digits. In a UK study of 485 patients with SLE, critical peripheral ischemia was seen in seven out of the 485 patients (1.4%). Vasculitis, which includes both small and large blood vessels, atherosclerosis, and microvascular thromboembolism are the main things that put people with SLE at risk for getting digital gangrene. The cutaneous type of vasculitis happens most often [11].

In juvenile SLE, there are two different categories of skin manifestations: one that is specific to SLE (facial malar rash, discoid lupus erythematosus (DLE)), and one that is not specific to the lupus type (cutaneous vasculitis). It frequently involves the upper and lower limbs, as we saw in our patient. Our patient had elevated C-reactive protein (CRP) at the initial presentation but had no history of Raynaud’s phenomenon. SLE can be associated with antiphospholipid antibodies (APLA), given the role pathophysiology of antiphospholipid syndrome (APS) plays in the development of digital gangrene. Antiphospholipid antibodies in APS syndrome contribute to microthrombus formation and subsequent digital gangrene. But this patient tested negative for APLA and lupus anticoagulant (LAC) antibodies. The cutaneous vasculitis can lead to petechiae, purpura, ulceration, necrosis, and gangrene, of which necrosis and gangrene result from decreased tissue perfusion. This is a small-vessel vasculitis mediated via immune complexes [12].

These immune complexes lodged on the basement membranes of the skin activate the complement system and cause inflammation. The cutaneous vasculitis can be a sole manifestation of SLE vasculitis or it can be part of the large multiorgan involvement. This patient also presented with symptoms such as fever and diffuse erythematous wheals on the face and body, especially on the back, prompting us to initially think it was urticaria. With digital ulceration and gangrene, urticaria is a rare feature of SLE, especially as a primary presentation. Urticaria was resolved after 24 hours of treatment with hydroxyzine. It is worth noting that SLE-related digital gangrene is more commonly seen in the adult age group. The occurrence of digital gangrene in pediatric age groups is rare, as in our case. A study involving 50 adult female SLE patients found that the individuals who developed cutaneous vasculitis were younger when compared to patients who did not develop vasculitis [13].

There are many drugs that can be used to treat SLE vasculitis and related digital gangrene. Some of these are corticosteroids, immunosuppressant drugs like mycophenolate mofetil [14], and monoclonal antibodies like rituximab. A study by Liu et al. looked at 2,684 SLE patients and found that Raynaud's phenomenon (RP), high serum C-reactive protein (CRP), and having the disease for more than four years all made it more likely that the patients would get digital gangrene. However, early treatment with steroids (prednisone ≥1 mg/kg/day started within three weeks) greatly lowered the risks of amputation [15].

The patient was prescribed prednisone (2 mg/kg/day) for the first three months. The prednisone dose was reduced gradually to 5 mg/day for the next three months, and the patient went into remission at the first follow-up with no recurrence of digital ulcers, urticarial rashes, or new manifestations.

## Conclusions

In conclusion, the presented case underscores the importance of recognizing atypical initial manifestations of systemic lupus erythematosus (SLE) in pediatric patients. Even though digital ulceration and gangrene are rare, they show how different SLE can look and how important it is to diagnose it early and treat it by a team of specialists to keep kids from getting worse and make sure they have the best possible outcomes.
